# An integrated intervention to reduce intimate partner violence and psychological distress with refugees in low-resource settings: study protocol for the Nguvu cluster randomized trial

**DOI:** 10.1186/s12888-017-1338-7

**Published:** 2017-05-18

**Authors:** Wietse A. Tol, M. Claire Greene, Samuel Likindikoki, Lusia Misinzo, Peter Ventevogel, Ann G. Bonz, Judith K. Bass, Jessie K. K. Mbwambo

**Affiliations:** 1 Department of Mental Health, Johns Hopkins Bloomberg School of Public Health, 624 N Broadway, HH863, Baltimore, MD 21205 USA; 2 Department of Psychiatry, Muhimbili University of Health and Allied Sciences, United Nations Road, Dar es Salaam, Tanzania; 3 United Nations High Commissioner for Refugees, Case Postale 2500, 1211 Geneva, CH Switzerland; 4 International Rescue Committee, 122 East 42nd Street, New York, NY 10168 USA

**Keywords:** Intimate partner violence, Mental health, Psychological distress, Refugees, Democratic Republic of the Congo, Tanzania, Cognitive behavioral therapy, Advocacy, Empowerment

## Abstract

**Background:**

Intimate partner violence (IPV) is a critical public health and human rights concern globally, including for refugee women in low-resource settings. Little is known about effective interventions for this population. IPV and psychological distress have a bi-directional relationship, indicating the potential benefit of a structured psychological component as part of efforts to reduce IPV for women currently in violent relationships.

**Methods:**

This protocol describes a cluster randomized controlled trial aimed at evaluating an 8-session integrated psychological and advocacy intervention (Nguvu) with female adult survivors of past-year IPV displaying moderate to severe psychological distress. Outcomes are reductions in: recurrence of IPV; symptoms of anxiety, depression and post-traumatic stress (primary); and functional impairment (secondary). Hypothesized mediators of the intervention are improvements in social support, coping skills and support seeking. We will recruit 400 participants from existing women’s support groups operating within villages in Nyarugusu refugee camp, Tanzania. Women’s groups will be randomized to receive the intervention (Nguvu and usual care) or usual care alone. All eligible women will complete a baseline assessment (week 0) followed by a post-treatment (week 9) and a 3-month post-treatment assessment (week 20). The efficacy of the intervention will be determined by between-group differences in the longitudinal trajectories of primary outcomes evaluated using mixed-effects models. Study procedures have been approved by Institutional Review Boards in the United States and Tanzania.

**Discussion:**

This trial will provide evidence on the efficacy of a novel integrated group intervention aimed at secondary prevention of IPV that includes a structured psychological component to address psychological distress. The psychological and advocacy components of the proposed intervention have been shown to be efficacious for their respective outcomes when delivered in isolation; however, administering these approaches through a single, integrated intervention may result in synergistic effects given the interrelated, bidirectional relationship between IPV and mental health. Furthermore, this trial will provide information regarding the feasibility of implementing a structured intervention for IPV and mental health in a protracted humanitarian setting.

**Trial registration:**

ISRCTN65771265, June 27, 2016.

**Electronic supplementary material:**

The online version of this article (doi:10.1186/s12888-017-1338-7) contains supplementary material, which is available to authorized users.

## Background

Intimate partner violence is a widespread and critical concern for human rights and public health globally. Intimate partner violence comprises physical, sexual, psychological, and/ or controlling behaviors, most commonly against women by their current or former male partners [1]. A recent synthesis of data from 141 studies in 81 countries found that globally 30.0% of women aged 15 years and older reported lifetime physical and/ or sexual intimate partner violence [2]. There is strong evidence for links between intimate partner violence and a range of negative outcomes for health and wellbeing in women, including mental health.

Consequences for women’s mental health include higher rates of depression, anxiety, posttraumatic stress disorder, and suicide [3, 4]. In the field of global health, there has been growing recognition of the importance of mental health concerns for population health, given their important contribution to the global burden of disease [5]. At the same time, researchers have been interested in the role of interpersonal violence as a social determinant of mental health in order to inform public mental health strategies [6, 7].

Intimate partner violence is also an acute concern in populations affected by armed conflict, the majority of which reside in low- and middle-income countries (LMIC). Few studies have been conducted with these populations, but a systematic review of 10 studies found that rates of intimate partner violence were particularly high in conflict-affected populations in LMIC – commonly much higher than rates of other forms of gender-based violence, e.g. sexual violence perpetrated by strangers [8]. Practitioners and researchers in settings of armed conflict have increasingly emphasized considering the importance of ongoing stressors such as intimate partner violence as determinants of mental health and psychosocial wellbeing, in addition to conflict-related events in the past [9, 10].

The current protocol describes a study evaluating an integrated intervention focused on both intimate partner violence and mental health. Study participants are refugee women from the eastern Democratic Republic of the Congo living in the Nyarugusu refugee camp in northwestern Tanzania. Refugees from the Democratic Republic of the Congo fled to Tanzania as a result of several decades of war which were characterized by widespread gender-based violence particularly in the eastern regions of North and South Kivu [11]. A recent survey found that two of every five women reported physical intimate partner violence, and one quarter reported ever experiencing sexual intimate partner violence [12]. These forms of violence have persisted as prevalent problems in Nyarugusu, which hosts over 60,000 Congolese refugees. In 2014, physical and emotional followed by sexual violence were the most commonly reported forms of intimate partner violence in this refugee camp [13]. Current estimates of the prevalence and incidence of intimate partner violence in Nyarugusu rely on passive ascertainment through reporting to protection agencies and are likely to underestimate the occurrence of intimate partner violence in the camp. Despite the lack of systematic monitoring data, intimate partner violence is recognized as one of the greatest protection concerns in Nyarugusu [13].

Not surprisingly, higher rates of mental health concerns have been identified in survivors of intimate partner violence in this population. A population-based study of adult women in the eastern Democratic Republic of the Congo found the estimated prevalence of past-year major depressive disorder (64.9%), past-month post-traumatic stress disorder (77.2%), past-year suicidal ideation (42.4%), lifetime suicide attempt (33.1%) and current substance misuse (20.5%) to be high among women with a history of intimate partner violence. With the exception of substance misuse, these estimates were significantly greater than the prevalence estimates of these disorders in women without a history of intimate partner violence [14].

Evidence on the effectiveness of efforts to prevent violence against women in LMIC overall is emerging [15]. Primary prevention efforts aim to reduce the number of new instances of intimate partner violence by addressing risk factors for violence, which commonly operate at multiple levels of the social ecology. Social, economic and combined interventions have been found to successfully reduce the incidence of intimate partner violence in LMIC [16]. Although these findings are promising, the current evidence suggests primary prevention efforts may have moderate effects, often on a select number of outcomes under investigation. In addition to primary prevention programming, secondary prevention may assist in stopping violence against women who are currently in an abusive relationship, or reducing chances for future victimization, e.g. when women in a violent relationship seek assistance in healthcare. For example, advocacy interventions commonly refer women to advocates who provide legal, housing, and financial advice; facilitate access to community resources; discuss safety planning; and provide ongoing support and counseling [17].

Although generic psychosocial support (e.g. generic supportive counseling) is a common element of advocacy interventions, there appears to have been little discussion regarding the contribution that more structured psychological support could make in strengthening the impact of secondary prevention interventions [17, 18]. Recent studies indicate that this may be a fruitful research direction, given identified bidirectional relationships between victimization and psychological distress consistent with a vicious cycle. For example, a large longitudinal study in South Africa found that intimate partner violence was associated with depressive symptoms, which in turn increased risk for future victimization [19]. Similarly, studies have found that posttraumatic stress disorder (PTSD) symptoms as a result of intimate partner violence may put women at increased continued risk for future violence [20, 21]. Further support for a bi-directional relationship is found in intervention research: reductions of PTSD symptoms in survivors of intimate partner violence through psychological intervention has been found to reduce risk for future victimization [22]. Research on this topic should be mindful of the risk of blaming women for the violence they endure (e.g., by pointing to individual factors such as psychological symptoms being associated with violence) [19]. For this reason, it is important to highlight: (1) the contextual factors that help perpetuate violence against women vis a vis factors at the individual level; (2) the importance of primary prevention interventions that may address such contextual factors; and (3) the observation that distress is a consequence of intimate partner violence before it contributes to further higher risk for violence. These critical issues notwithstanding, the existing findings that indicate a vicious cycle between intimate partner violence and psychological distress lend support to the hypothesis that multi-component interventions may play a role as part of the comprehensive efforts required to reduce intimate partner violence. Interventions that simultaneously empower women through an advocacy component, as well as reduce psychological distress through structured psychosocial interventions, may have stronger secondary preventive effects than advocacy interventions that do not address psychological distress in a structured manner.

Against this background, this study protocol describes a cluster randomized trial of a secondary prevention intervention that integrates (1) an advocacy intervention with (2) a structured psychosocial support component, aimed at reducing both intimate partner violence and psychological distress in women currently in violent relationships as compared to standard services available in Nyarugusu camp. A cluster design with local women’s groups as clusters was preferred to capitalize on an existing infrastructure of women’s groups operating within villages in the camp, which was evaluated as an accessible yet safe structure for recruitment.

## Methods/design

The manuscript reports the study protocol in accordance with the CONSORT guidelines for cluster randomized trials and the SPIRIT guidelines for intervention trial protocols (checklists included as Additional files 1 and 2).

### Objectives

The aim of this cluster randomized trial is to evaluate the efficacy of an integrated mental health and advocacy intervention named Nguvu (KiSwahili for strength) for improving psychosocial health and reducing the recurrence of intimate partner violence among Congolese refugee women in Nyarugusu Refugee Camp, Tanzania. We hypothesize that women in the intervention condition (consisting of the 8-session Nguvu intervention and access to intervention as usual), relative to intervention as usual, will report fewer incidents of physical, sexual, and psychological intimate partner violence and experience less psychological distress (primary outcomes) as well as reduced levels of functional impairment (secondary outcome).

**Fig. 1:**
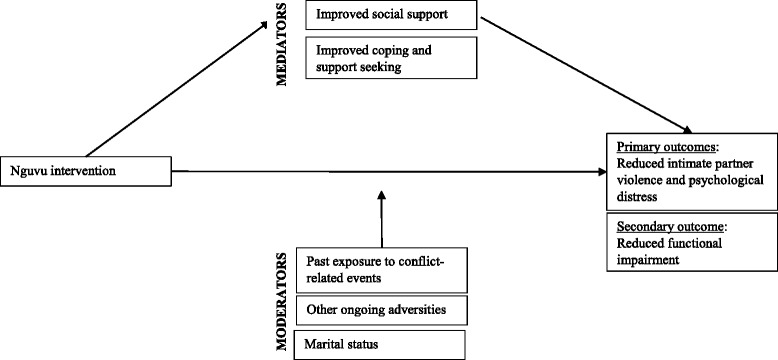
Hypothesized mediators and moderators of intervention

In addition, we will assess mediators and moderators of the intervention (Fig. 1). In terms of mediators, we hypothesize that the intervention will increase social support and lead to stronger use of coping skills and support seeking. We expect that these improvements will in turn be associated with reductions in psychological distress, reports of intimate partner violence, and levels of functional impairment. We expect there will be other (unmeasured) factors that explain changes on outcome measures, so we hypothesize a partial mediation effect. With regard to moderators, we anticipate that women who have experienced fewer other potentially traumatic events, and who currently face fewer ongoing adversities, will derive greater benefits from the intervention. All outcomes of interest will be assessed at the participant level, as opposed to the cluster (women’s group) level.

### Setting

Nyarugusu is a refugee camp located in northwest Tanzania in the Kasulu district, Kigoma Province. The camp hosts over 60,000 Congolese refugees. Many of the Congolese residents arrived at the camp in 1996 following the war in South Kivu and have thus lived in Nyarugusu for two decades [1]. Administrative units in the camp are divided into zones, villages and clusters. Each cluster contains 60–100 plots, each of which are allocated to a family [23, 24]. Historically, Nyarugusu has consisted of seven zones subdivided into 52 villages. In April 2015, a large influx of refugees from Burundi (around 140,000 refugees as of June 2016) prompted the expansion of Nyarugusu into 11 zones [25]. The four recently added zones were developed as temporary and primarily served Burundian, as opposed to Congolese, refugees. Within the current project we do not focus on the Burundian refugee population, as the project was initiated when Congolese refugees made up 94% of the Nyarugusu camp. Resources for development and adaptation of the intervention in multiple languages were not anticipated.

### Participants and recruitment

*Randomization*: Clusters will be made up of local women’s groups. We will randomize women’s groups in zones 2 through 7 in Nyarugusu to intervention and control conditions, based on a list supplied by a humanitarian agency. There are 63 women’s groups located within 31 of the 46 villages in zones 2 through 7. Other zones (8 through 11) are inhabited mainly by Burundian refugees. Thus all women that are members of these women’s groups and meet study eligibility criteria in zones 2 through 7 may be included in the trial. Staff not working for the project at Johns Hopkins Bloomberg School of Public Health will randomize 32 women’s groups into the intervention and 31 women’s groups into the control condition, using a random number generator in Stata version 14. The allocation of women’s groups to study conditions will be kept concealed from the local research team, and will be shared with the intervention teams only immediately before planning intervention implementation.

*Sample Size*: With projected attrition of 20% we require 400 women meeting eligibility criteria to be enrolled in this study in order to achieve ≥80% power to detect an effect size of 1.6 for depression/anxiety (measured using the Hopkins Symptom Checklist) and 1.3 for post-traumatic stress symptoms (measured using the Harvard Trauma Questionnaire). These effect sizes represent the between-group differences in the change from baseline to the final follow-up assessment in the two primary mental health outcome measures. Given that the intra-cluster correlation coefficient (ICC) and the within-subject correlation in our outcome measures over time is unknown, a sample size of 200 women per intervention condition would allow us to retain 80% power with varying ICC values (range: within-subject ICC: 0.1–0.5; within-cluster ICC: 0.1–0.3). Parameters used to produce these sample size calculations were based on results from a previous cluster randomized controlled trial of Cognitive Processing Therapy employing these measures in a population of Congolese female survivors of sexual violence [26].

*Recruitment*: Individual participants will be recruited through local women’s groups. Women’s groups have, on average, 16.6 women per group (SD = 7.3). Women’s groups have been organized in the camp by the United Nations High Commissioner for Refugees (UNHCR) implementing partners to provide skills training and an opportunity for women to strengthen their social networks. We intentionally selected a sample population that could be recruited and contacted within a pre-existing structure that was only available to women in order to protect women from potential harm resulting from their partner’s (or others’) awareness of their participation in a study focused on intimate partner violence.

Recruitment will take place in two steps. In the first step the research team leader will contact the women’s group leaders and ask for their permission to present a brief summary of the program to women in their group. Upon receiving permission to approach women participating in groups at least two research assistants will present a scripted summary of the program that describes the study as a women’s health intervention and offers to provide more specific information to women through an informed consent and screening process if they are interested.

*Eligibility and screening*: We will apply the following inclusion criteria for participation in the study: (1) Women; (2) 18 years of age or older; (3) married, living with a partner, or in a relationship in the last 12 months; (4) a refugee from the Democratic Republic of the Congo residing in Nyarugusu camp; (5) current member of a registered local women’s group; (6) screened positive for a past-year history of intimate partner violence (physical or sexual); and (7) screened positive for a moderate or severe level of psychological distress. Exclusion criteria will include: (1) current imminent risk of suicide; and (2) observable signs of severe (neuro)psychiatric disorder that impedes participation in a group intervention (e.g. acute psychosis; severe substance misuse). Women at immediate physical risk of injury from intimate partner violence and other forms of protection risks will be included in the study and will be linked to protection services as part of standard operating procedures in Nyarugusu.

To assess eligibility, research team members will request oral consent for screening from individual participants and subsequently administer a brief demographic inventory including questions about age, refugee status and partnership status, the 5-item Abuse Assessment Screen (AAS) [27], the 25-item Hopkins Symptom Checklist (HSCL-25) [28, 29], and the 16-item Harvard Trauma Questionnaire (HTQ) [30]. To be eligible for the study, women must indicate an average score ≥ 1.75 on the HSCL-25 or >1.0 on the HTQ, which have been used as indicators of significant psychological distress (i.e., depression, anxiety, and PTSD complaints respectively) in prior research with women in eastern DRC [26]. In addition to reporting significant psychological distress, women will be included if they have experienced physical and/or sexual intimate partner violence as assessed with the Swahili version of the AAS [31]. Women meeting all eligibility criteria will be approached for consent to a baseline (T1) assessment with a member of the research team that includes the other measures. Exclusion criteria will be assessed using the HSCL-25, a suicide risk assessment and a 6-item assessor-reported measure of signs of severe mental illness that will impede participation in a group intervention (e.g. psychosis, severe substance misuse). A single item on the HSCL-25 assesses suicidal ideation (In the past 4 weeks how often have you experienced the problem of thoughts of ending your life; not at all, a little bit, moderate amount, or a lot). If a participant endorses any suicidal ideation (a little bit, moderate amount, or a lot) they will proceed to complete a full suicide risk assessment module. Participants meeting criteria for imminent or impulsive risk of suicide will be excluded from the study and a safety protocol will be initiated that includes contacting a supervisor and not leaving the participant alone until they meet with a counselor. An assessor-reported measure of severe mental illness will inquire about abnormal or disorganized behavior, delusions, hallucinations, manic symptoms and severe substance use disorder. If the observer believes that participant meets the aforementioned criteria, they will be excluded from the study.

#### Procedures

The research team in Nyarugusu consists of one research team leader and nine research assistants (interviewers). All research assistants are Congolese refugee women. The principal investigator trained the research team leader in a three-day workshop covering topics related to research methods and management. The research team received a 10-day training in research methods which was primarily composed of practice-based training with some supplemental didactic instruction on the following topics: roles and expectations of the research team, project overview, methodological considerations (e.g. validity and potential biases, reliability), recruitment procedures, data collection procedures, data management, research ethics and informed consent, and retention strategies (e.g. maintaining contact, safe contact methods). During this training, the research team administered mock assessments, which were observed by trainers and other trainees.

**Fig. 2:**
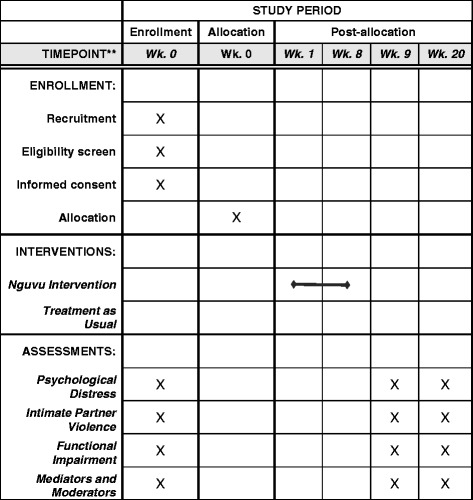
Schedule of enrollment, interventions, and assessments

All assessments will be conducted in a private location of the preference of the participant, either the Empowerment Center, community centers, health centers, or another location. The research assistants will be masked to the participant’s study assignment. It is possible that a research assistant may become unmasked by information the participant divulges during the assessment; however, research assistants are trained not to inquire about study assignment and participants are asked not to disclose this information to members of the research team. We do not anticipate intentionally needing to unmask the allocated study condition for a given participant to the research assistants during the trial. After the baseline assessment a list of study IDs and women’s group membership for each enrolled participant will be provided to the research team leader. The research team leader will share the contact information for participants that are members of groups randomized to Nguvu intervention facilitators to schedule the first Nguvu intervention session (Fig. 2). The research team leader is not masked to intervention assignment; however, she does not participate in data collection and is instructed to keep women’s group allocation concealed from the research assistants.

#### Intervention

*Formative research*: We engaged in a period of qualitative research to select and adapt existing evidence-based interventions. This qualitative research included (1) a preliminary site visit; (2) a desk review of existing academic and grey literature with relevance to gender-based violence and mental health [32]; (3) rapid qualitative research (free listing and key informant interviews); and (4) formation and engagement with a community advisory board. First, in a preliminary site visit (WT, SL), meetings were organized with key stakeholders, including the UNHCR, UNHCR implementing agencies for health and protection (Tanzania Red Cross Society and International Rescue Committee respectively), and refugee incentive workers engaged in protection activities. In these meetings we discussed the potential for a project focused on gender-based violence and mental health, and needs of survivors of gender-based violence in Nyarugusu. Second, we conducted a desk review summarizing academic and unpublished literature on gender-based violence and mental health with a focus on residents of and refugees from the eastern DRC. We also gathered information about gender-based violence, mental health and existing resources for these issues in Nyarugusu camp. The desk review is publicly available and can be found at http://mhpss.net/resource/mental-health-and-psychosocial-wellbeing-in-congolese-refugee-survivors-of-gender-based-violence/.

The preliminary site visit and desk review revealed intimate partner violence to be the most common form of gender-based violence in the camp. Intimate partner violence was perceived to be associated with significant psychological distress, while there being few structured services available to reduce distress associated with intimate partner violence. Third, we conducted free listing interviews (*n* = 40) and key informant interviews (*n* = 15) with refugees working with humanitarian agencies in the camp in order to assess refugee perspectives with regard to (a) the most common psychological problems; (b) which groups were most affected by these problems; and (c) what types of services and supports were available. Upon completion of the free listing interviews, the symptoms were ranked with respect to frequency and saliency. The three most salient problems affecting women affected by intimate partner violence were ‘stress’ (*msongo wa mawazo*), ‘sadness’ (*huzuni*) and ‘fear’ (*hofu*). Information from the formative phase of the research was shared with the Nguvu community advisory board for further discussion and assistance with interpretation.

Findings from the formative research phase indicated that an intervention focused on psychological distress more broadly (as opposed to a focus on one specific mental disorder, such as posttraumatic stress disorder or major depressive disorder) would be more helpful. Building on this information, we searched for evidence-based interventions that have been found to effectively reduce psychological distress in gender-based violence affected populations, and intimate partner violence, in low-resource settings. In addition, we selected reliable and valid measures that have measured psychological distress in conflict-affected populations (Table 1). These measures were evaluated through a pilot study of 60 women in Nyarugusu meeting eligibility criteria for the randomized controlled trial (RCT). As part of this pilot study we assessed inter-rater reliability, test-retest reliability, construct validity and internal consistency. Each pair of facilitators completed one course of the intervention with 12 pilot participants per pair to pilot implementation of the intervention under study.

**Table 1:** Overview of Measures

Construct	Measure	# items	Assessments			
Screening & Primary outcomes			Screening (T0; Week 0)	Baseline (T1; Week 0)	Post-Intervention (T2; Week 9)	3-Month Follow-up (T3; Week 20)
Psychological Distress	Hopkins Symptom Checklist 25–Anxiety subscale	10	X		X	X
	Hopkins Symptom Checklist 25 – Depression subscale	15	X		X	X
	Harvard Trauma Questionnaire – Part 4	16	X		X	X
Intimate Partner Violence	Abuse Assessment Screen	5	X			
	Demographic and Health Survey Domestic Violence Module	21		X	X	X
Secondary outcomes						
Functional impairment	Items developed in South Kivu and adapted in Nyarugusu during pilot study	12		X	X	X
Mediators and moderators						
Socio-demographics	Locally developed (Screening)	6	X			
	Locally developed (Baseline)	16		X		
Suicide Risk Assessment	Based on WHO mhGAP procedures	3	X		X	X
Serious Mental Illness	Based on WHO trial procedures	5	X			
Trauma and major life events	Harvard Trauma Questionnaire – Part 1; Items developed in South Kivu	25		X	X	X
Coping and service use	Items developed in South Kivu	13		X	X	X
Structural social capital	Items developed in South Kivu	17		X	X	X
IPV safety planning	Safety-Promoting Behavior Checklist	13		X	X	X

*Development of the Nguvu Intervention*: A previous systematic review identified only one randomized controlled trial of a mental health and psychosocial support intervention for gender-based violence affected populations in the context of armed conflict in a low-resource setting [33]. This trial focused on Cognitive Processing Therapy, a type of cognitive behavioral therapy with a trauma focus, and found it to be highly effective in reducing symptoms of depression, anxiety, and posttraumatic stress disorder, as well as in reducing functional impairment [26]. Given that this intervention was implemented in South Kivu, Democratic Republic of the Congo, we decided to adapt this intervention for the Congolese refugee population in Nyarugusu. Cognitive behavioral therapy with a trauma focus is recommended by the World Health Organization for management of posttraumatic stress disorder in non-specialized low-resource settings (e.g. primary care and community settings in low- and middle-income countries) [34]. However, there are major challenges to implementing this recommendation, including a lack of trained human resources and high demands on staff in non-specialized settings [35]. To reduce pressure on limited human resources and their available time, we were interested in shortening the 12-session cognitive processing therapy protocol evaluated in the Democratic Republic of the Congo. A previous protocol of 6 sessions had been developed for women recently confronted with gender-based violence [36, 37].

In addition, we were interested in identifying an evidence-based approach to advocacy and empowerment. To our knowledge, the only randomized controlled trial showing impacts of an advocacy intervention for survivors of intimate partner violence in a non-Western setting is the work published by Tiwari and colleagues in Hong Kong [38].

*Structure of the Nguvu intervention*: We worked with the lead authors of the three intervention manuals (the 12- and 6-session Cognitive Processing Therapy manuals; the advocacy manual) to develop an 8-session intervention called Nguvu (KiSwahili for strength). The result is an 8-session intervention consisting of one individual initial session, followed by seven group sessions, delivered once per week over 8 weeks in total (see Table 2). Refugee incentive workers in Nyarugusu have been trained to deliver the intervention by two expatriate trainers and a Tanzanian clinical psychologist, leading to further adaptations in the intervention manual.

**Table 2:** Structure of the Nguvu Intervention

Session	Topic	Description	Homework Activities
1. Empowerment/ Advocacy	Advocacy and safety plan	• Information on IPV • Discussing psychological distress • Danger assessment • Safety plan and emergency plan	Safety plan
2. CPT	Intro to CPT in Nguvu	• Introducing group rules and overview of Nguvu sessions • Stuck thoughts • Explanation of thoughts and feelings • Treatment goals	Notice and explore thoughts and distress related to IPV
3. CPT	ABCs	• Introduction of ABCs • Exploring stuck points • Group relaxation	Daily practice of ABCs and relaxation task
4. CPT	Stuck points and thinking questions	• Changing thoughts and feelings • Thinking questions • Exploring stuck thoughts	Daily practice of ABCs and exploration of stuck thoughts; relaxation task
5. CPT	Learning safety and trust	• Introduction to safety and trust • Stuck thoughts related to trust	Daily practice of ABCs, thinking questions and changing thoughts
6. CPT	Power, control and self-esteem	• Introduction to power/control issues related to self and others • Challenging questions for control issues • Self-esteem • Caring related to self and others	Daily practice of ABCs, thinking questions and changing thoughts; self-care
7. CPT	CPT Review	• Discuss the impact of distressing events • Planning for the future	Daily practice of ABCs, thinking questions and changing thoughts; self-care; revise safety plan; relaxation exercise
8. Empowerment/ Advocacy	Review of advocacy and safety plan	• Review safety plan • Advocacy • Coping and support methods	--

The Nguvu intervention begins with an individual session with a participant and a facilitator that is intended to present the expectations for the intervention, begin a discussion surrounding intimate partner violence and psychological distress, conduct a thorough danger assessment reviewing the intimate partner violence situation of the participant, and develop a realistic safety plan. The content of this intervention is based on previous research on advocacy interventions for survivors of intimate partner violence [39] . Sessions two through seven, based on Cognitive Processing Therapy, develop the participant’s skills and ability to identify and understand the relationship between a potentially traumatic event, thoughts and feelings. These sessions encourage restructuring of ones thoughts such that the participant may modify their feelings in a positive manner. These sessions were based on the cognitive processing therapy manual evaluated in the eastern DRC in survivors of sexual and gender-based violence [26]. The final session is a group advocacy session that revisits the safety plans developed in session one and provides a forum for discussion on how to improve one’s safety plan and share safety and coping strategies that have been effective for other group members. A thorough description of the content of each session is described in Table 2.

*Training, supervision and delivery of the intervention*: Ten Congolese refugees working as lay psychosocial workers in Nyarugusu camp for the International Rescue Committee (IRC) gender-based violence and women’s empowerment programs were selected as facilitators for the intervention. The facilitators received 9 days of training that covered basic counseling skills, the intervention manual and self-care strategies.

As part of the trial, facilitators will be expected to deliver 1–5 sessions of the intervention per week depending on the number of active groups (1 session per group per week). A single facilitator delivers individual sessions (Session #1), while a pair of facilitators delivers the group sessions (Sessions #2–8). The facilitators will receive ongoing support from a psychologist on site. In addition, facilitators will receive weekly telephone supervision by the two clinical psychologists who trained facilitators in the intervention, one of which is based in Tanzania and will make periodic trips to the study site to conduct in-person supervision. The facilitators will also conduct weekly peer supervision and self-care sessions organized by the lead facilitator. During supervision, complex cases will be discussed and the facilitators, together with their supervisors, will use clinical judgment to determine if such cases should discontinue participation and whether a referral to other services may be needed. The research team leader using will monitor intervention fidelity and participant attendance.

#### Intervention as usual

The intervention as usual control condition will have access to standard mental health and protection services during the trial period. There are two types of protection activities in Nyarugusu that focus on preventing and responding to intimate partner violence respectively. The first (the prevention focused activities) consist of awareness raising activities and trainings to educate the community about intimate partner violence and gender-based violence more broadly. The response program constitutes the services available for persons affected by intimate partner violence. In the camp there are two support centers where refugees may report incidents of intimate partner violence. Survivors of intimate partner violence are assigned to a caseworker and may then be provided psychosocial counseling and support, non-food items, legal counseling and support, and referrals to other protection, medical or legal services [1]. Existing psychosocial services include the following activities: 1) providing psychological first aid and basic counseling; 2) supporting social reintegration, vocational training and empowerment; and 3) referral to mental health services as necessary. The response program will function as the intervention as usual condition.

#### Outcome assessments

Unaware of study assignment, the research team will interview women in the week before the start of the intervention, 1 week after the intervention period (i.e., approximately a 9-week interval) and 12 weeks after the intervention. The measures and interview guides are translated and administered in Kiswahili at the individual participant level.

*Primary outcomes*: An overview of measures is provided in Table 1. The primary outcomes of interest are psychological distress and intimate partner violence. Psychological distress will be measured using the 25-item Hopkins Symptom Checklist (HSCL-25; [29]) and Part 4 of the Harvard Trauma Questionnaire (HTQ; [39]). Items included in the HSCL-25 assess depressive and anxiety symptoms. Items on the HTQ assess posttraumatic stress symptoms. Intimate partner violence will be measured using the Demographic and Health Survey Domestic Violence Module, which is adapted from items on the Conflict Tactics Scale [40, 41].

*Secondary outcomes*: Functional impairment, measured using items developed through qualitative research, will serve as the secondary outcome in this study.

*Mediators and moderators*: Mediators included in this study include social support, coping and service utilization. Moderators measured in this study include previous exposure to potentially traumatic events, ongoing potentially traumatic events and marital status (Fig. 1). Mediators and moderators were measured using relevant modules from the trial on Cognitive Processing Therapy in the Democratic Republic of the Congo [26].

Kiswahili versions of the aforementioned measures were adapted through an iterative translation and adaptation workshop whereby research assistants, team leader and trainers piloted the measures and collaboratively identified and revised terminology or phrasing that was not comprehensible or consistent with the Kiswahili spoken in Nyarugusu camp. In situations where only an English version of the measure was available, the research team leader, a native Tanzanian Kiswahili speaker, conducted the initial translation and then proceeded with the translation and adaptation workshop involving the local research team as described above.

#### Analysis

This study employs a longitudinal, cluster-randomized controlled trial of two groups with randomization occurring at the cluster (i.e. women’s group) level and analyses occurring at the individual (i.e. participant) level. Descriptive analyses will estimate means, proportions and variability of all measures in the experimental and control condition. We will evaluate whether any participant baseline characteristics are associated with attrition to inform the strategy for managing missing data in analysis of our primary and secondary outcomes. Variables associated with attrition will be included as covariates and/or incorporated into the multiple imputation model to account for missing data.

Analysis of the primary and secondary outcomes, symptoms of depression, anxiety, post-traumatic stress, intimate partner violence and functional impairment, will be evaluated using linear mixed-effects models. The basic model will include a random intercept for women’s group and participant and fixed effects for study condition (intervention vs. control), time (in weeks), an interaction between condition and time, and factors related to attrition. The effect of interest is the interaction between condition and time, which captures between-group differences in the trajectory of the outcome over the follow-up period. We will then evaluate, based on visual inspection of the data and model fit, whether the inclusion of random slopes for cluster and participant is appropriate. Moderators of treatment effects will be included as 2- and 3-way interactions (e.g. marital status x time, marital status x intervention, marital status x time x intervention). Mediation will be assessed by evaluating attenuation in the effect of intervention on the outcomes of interest at T3 upon addition of the mediator (measured at T2) as a covariate in the previously described models. All participants enrolled in the study will be included and analyzed with respect to their original treatment assignment as per intention-to-treat (ITT) principles.

#### Data management

Data collection forms will be kept in a locked safe temporarily at the study field site until collected by the research team leader and stored in a locked cabinet at the study management office in Kasulu. All data will be double entered using a codebook into Excel. Throughout data collection, the database will be periodically checked for outliers that may fall beyond the range of possible values and other indications of data entry errors.

#### Ethics

All participants will go through an informed consent process whereby the research assistant explains the study to the potential participant and requests written informed consent if she is interested in participating. If informed consent is obtained the research assistant proceeds to orally administer all measures. Participant data will remain confidential and will not be shared beyond the Nguvu investigators and research staff. Any data shared outside of the immediate research team upon completion of the study will be completely de-identified. Sharing this data and other study-related documents will be at the discretion of the Principal Investigator.

The Institutional Review Boards of Johns Hopkins Bloomberg School of Public Health (IRB0007219) and Muhimbili University of Health and Allied Sciences (MUHAS; 2014–10-27/AEC/Vol.X/56) have approved and continue to monitor the trial, including review of any future protocol amendments. Any serious or unanticipated adverse events and significant protocol deviations will be reported to the aforementioned Institutional Review Boards. The National Institute of Medical Research in Dar es Salaam, Tanzania also approved this study (NIMR/HQ/R.8a/Vol.IX/2016). A Data and Safety Monitoring Board (DSMB) consisting of researchers from MUHAS with expertise in the conduct of RCTs was organized to monitor early intervention effects with the intention of ensuring ethical implementation of the trial. An independent, masked statistician will evaluate intervention effects on the primary outcomes when half of the participants have completed the post-intervention assessments. The unmasked DSMB will evaluate the results and recommend premature termination of the study if there is strong statistical evidence (e.g. symmetrical stopping boundaries at *p* < 0.001) of intervention effects. In addition, a community advisory board was assembled by the research team to represent local leaders in Nyarugusu camp with experience working in the area of gender-based violence. The research team will continue to hold regular meetings with the community advisory board to review proposed intervention strategies, research procedures and overall progress. The community advisory board serves as the liaison between Nguvu staff. Upon completion of the trial, results will be disseminated to the community via the community advisory board. Dissemination of results through presentations, workshops (locally, nationally, internationally), humanitarian newsletters and websites, and publications are also planned and will adhere to recommended authorship guidelines [42]. We plan for a specific workshop with the International Rescue Committee to plan for sustainable integration of the intervention in humanitarian services, if proven effective.

#### Collaboration and funding

This study is a collaboration between Johns Hopkins Bloomberg School of Public Health, the United Nations High Commissioner for Refugees, Muhimbili University of Health and Allied Sciences and the International Rescue Committee. It is funded by Research for Health in Humanitarian Crises (R2HC), a program co-funded by United Kingdom Department for International Development (DFID) and the Wellcome Trust. Johns Hopkins University is the overall coordinating center for the trial. Muhimbili University of Health and Allied Sciences is responsible for data collection and management, and the International Rescue Committee coordinates intervention activities.

## Discussion

This cluster randomized trial aims to evaluate the impact of a secondary prevention intervention with Congolese refugee women in Nyarugusu refugee camp in Tanzania. Similar to existing findings with conflict-affected populations, formative research found intimate partner violence to be a critical health issue in the refugee camp, with few resources to address its mental health and psychosocial impacts. The intervention combines a short evidence-based psychotherapy (Cognitive Processing Therapy) with an advocacy intervention, both previously evaluated as efficacious in low-resource settings. Primary outcomes are a reduction in psychological distress, and reduced exposure to intimate partner violence. Given previously identified bi-directional relationships between intimate partner violence and psychological distress, an integrated intervention has the potential to break the vicious cycle between intimate partner violence more effectively than single component interventions.

We purposely planned an evaluation of a time-limited intervention in a real-world refugee setting context, through partnership with humanitarian agencies active in the camp (UNHCR, IRC), using humanitarian intervention infrastructure available in the camp setting. This research approach was intended to facilitate easier dissemination and implementation of study findings, which has been a major challenge for currently recommended evidence-based interventions for conflict-affected populations in low-resource settings [35, 43]. If proven effective, the intervention could have important implications for humanitarian health programming in low-resource refugee settings.

There are several limitations that need to be taken into account when evaluating the potential impact of the study findings. First, the trial evaluates the impact of an integrated intervention compared to care as usual in a two-armed trial. It will therefore not be possible to isolate the specific effects of either the psychological or intimate partner violence components. However, both components have been individually tested in randomized controlled trials in low-resource settings. In our view it is currently a higher priority to evaluate the potential impacts of an integrated package, so that future research can focus on teasing apart the impacts associated with different modules in more complex designs.

Second, assessing generalizability of study findings is complex. On the one hand, we purposely selected a site that represents a common refugee scenario (i.e., protracted refugee setting, refugees displaced within the developing world, high levels of intimate partner violence). However, the unique dynamics of this particular refugee camp (e.g., co-occurring influx of Burundian refugees, continuing tensions in the eastern DRC, an ongoing resettlement process for Congolese refugees) caution against wide-ranging applicability of study findings to other populations. If results are positive, the next step in development of this integrated intervention would be replication of its impacts in other protracted refugee settings in low- and middle-income countries, as well as in other types of settings.

Third, we are evaluating a shorter version of the psychotherapeutic component than was evaluated previously in the eastern DRC. It may be that this negatively impacts the effect size. However, we also added an intimate partner violence intervention component, which formative research indicated is a critical contributor to psychological distress for women in this refugee camp. This additional component has the potential to increase impact of the intervention. In addition, our sample size calculations allow for different scenarios with regard to size of intra-cluster correlation and effect size, so that we can be reasonably confident that the study is sufficiently powered.

Despite these limitations, we believe this evaluation has the potential to provide important information on two vital inter-connected health issues for refugees, i.e. mental health and intimate partner violence, on which currently little is known with regard to effective intervention options. The evaluation of an intervention delivered in a dynamic and complex refugee setting, building on resources available with humanitarian agencies in a low-resource context, represents a higher risk strategy that can deliver highly relevant results for humanitarian practice.

## Trial status

At the time of manuscript submission, the pilot study had been completed and recruitment for the full trial had not started.

## Additional files

Additional file 1: Table S1. CONSORT 2010 checklist. (DOCX 31 kb)

Additional file 2: SPIRIT 2013 checklist. (DOC 122 kb)

## Abbreviations

AAS: Abuse Assessment Screen
DFID: Department for International Development
DRC: Democratic Republic of the Congo
DSMB: Data and Safety Monitoring Board
GBV: Gender-based violence
HSCL-25: Hopkins Symptom Checklist (25-item)
HTQ: Harvard Trauma Questionnaire
ICC: Intra-cluster correlation coefficient
IRC: International Rescue Committee
ITT: Intention-to-treat
LMIC: Low- and middle-income country
MUHAS: Muhimbili University of Health and Allied Sciences
PTSD: Post-traumatic stress disorder
R2HC: Research for Health in Humanitarian Crises
RCT: Randomized controlled trial
UNHCR: United Nations High Commissioner for Refugees
